# Proposing a Core Outcome Set for Physical Activity and Exercise Interventions in People With Rare Neurological Conditions

**DOI:** 10.3389/fresc.2021.705474

**Published:** 2021-10-21

**Authors:** Gita Ramdharry, Valentina Buscemi, Annette Boaz, Helen Dawes, Thomas Jaki, Fiona Jones, Jonathan Marsden, Lorna Paul, Rebecca Playle, Elizabeth Randell, Michael Robling, Lynn Rochester, Monica Busse

**Affiliations:** ^1^National Hospital for Neurology and Neurosurgery, University College London Hospitals NHS Trust, London, United Kingdom; ^2^Institute of Neurology, University College London, London, United Kingdom; ^3^Faculty of Health, Social Care and Education, St. George's, University of London, London, United Kingdom; ^4^Faculty of Health and Life Sciences, Oxford Brookes University, Oxford, United Kingdom; ^5^Medical Research Council Biostatistics Unit, University of Cambridge, Cambridge, United Kingdom; ^6^Department of Mathematics and Statistics, University of Lancaster, Lancaster, United Kingdom; ^7^Faculty of Health, Social Care and Education, Kingston University, Surrey, United Kingdom; ^8^Faculty of Health, Plymouth University, Plymouth, United Kingdom; ^9^Department of Physiotherapy and Paramedicine, Glasgow Caledonian University, Glasgow, United Kingdom; ^10^Centre for Trials Research, Cardiff University, Cardiff, United Kingdom; ^11^Institute of Neuroscience, Newcastle University, Newcastle, United Kingdom

**Keywords:** physical activity, neuromuscular disease, motor neurone disease, Huntington's disease, inherited ataxias, hereditary spastic paraplegia, parkinsonism, outcome measurement instruments

## Abstract

Rare neurological conditions (RNCs) encompass a variety of diseases that differ in progression and symptoms but typically include muscle weakness, sensory and balance impairment and difficulty with coordinating voluntary movement. This can limit overall physical activity, so interventions to address this are recommended. The aim of this study was to agree a core outcome measurement set for physical activity interventions in people living with RNCs. We followed established guidelines to develop core outcome sets. Broad ranging discussions in a series of stakeholder workshops led to the consensus that (1) physical well-being; (2) psychological well-being and (3) participation in day-to-day activities should be evaluated in interventions. Recommendations were further informed by a scoping review of physical activity interventions for people living with RNCs. Nearly 200 outcome measures were identified from the review with a specific focus on activities or functions (e.g, on lower limb function, ability to perform daily tasks) but limited consideration of participation based outcomes (e.g., social interaction, work and leisure). Follow on searches identified two instruments that matched the priority areas: the Oxford Participation and Activities Questionnaire and the Sources of Self-Efficacy for Physical Activity. We propose these scales as measures to assess outcomes that are particularly relevant to assess when evaluating physical activity interventions mong people with RNCs. Validation work across rare neurological conditions is now required to inform application of this core outcome set in future clinical trials to facilitate syntheses of results and meta-analyses.

## Introduction

Rare neurological conditions (RNCs), where cases are ≤40 per 100,000 population (1), collectively incur a significant cost burden to healthcare, social care services and informal care (2). Despite variability across conditions, many of these conditions will share symptoms and signs at the level of body function, activities and participation (3). As such, common approaches to improve fitness (e.g., cardiovascular and strength training), activity (e.g., balance and gait training) and participation levels (e.g., supported self-management) are often implemented in clinical practice (4–8).

Physical activity is any bodily movement produced by the muscles that require us to expend energy and can include structured exercise, active transportation, household chores, and activity during work, play and recreation (9). Whilst trials of physical activity interventions in RNCs highlight the potential of physical activity interventions to improve fitness and function (10–13) they are often small studies that fail to influence clinical practice. There are a variety of factors that limit the impact of these trials, not least the selection of outcome measures. Measurement constructs may vary from physiological measures (e.g., strength and fitness), to functional assessments (e.g., walking speed, climbing stairs) or quality of life and well-being outcomes and do not typically take into account patient preferences (14). If we are to ensure that research is relevant and able to influence clinical practice and future research, we need to ensure the use (and reporting) of standardized, relevant outcome measures within the field that are applicable to people living with the conditions (15, 16). Importantly, it should not be assumed that measurement should be restricted to the agreed core outcomes but rather that these outcomes should always be gathered and reported to facilitate evidence synthesis across relevant trials and studies. Core outcome sets have been agreed for specific target conditions for example cancer, rheumatology and chronic pain as well as for specific care pathways for example maternity care (17, 18).

Core outcome sets have also been proposed for people with neurologic conditions (19), including adults with dementia (20). However, these core sets have not been tailored to physical activity interventions for people living with RNCs. Recommending measures would not only help to bring consistency in reporting, allowing comparisons or meta-analysis of future studies, but also ensure responses are measured of constructs important to people living with RNCs. This study focuses on the development of an agreed standardized set of outcomes termed a “core outcome set” (21) that should be at a minimum measured and reported in trials of physical activity interventions in people living with RNCs.

## Method

The study followed the guidelines of the COSMIN (COnsensus-based Standards for the selection of health Measurement INstruments) and COMET (Core Outcome Measures in Effectiveness Trials) initiatives. A four step approach was used to select outcome measurement instruments recommended within core outcome sets (21). The activities relevant to each step within the process are described in detail below.

We focused on groups of progressive RNCs, namely neuromuscular diseases, Ataxias, Huntington's Disease (HD), Atypical Parkinson Diseases (AP), including Progressive Supranuclear Palsy, Multiple Systems Atrophy, Corticobasal Degeneration, Motor Neuron Diseases (MND) and Hereditary Spastic Paraparesis (HSP). These conditions affect ~2–10 per 100,000 in the general population, collectively leading to limited mobility and poor balance for many individuals. People with neuromuscular diseases and MND experience profound weakness and muscle atrophy. People with Ataxia, HD, AP, and HSP experience difficulty controlling movement, with some muscle weakness and variable cognitive impairment. Many people across the conditions also experience pain, joint deformity, fatigue and depression which impacts on their ability to participate in routine activities of daily living.

### Step One: Conceptual Considerations

People living with RNCs, carers of people with RNCs and representatives from five collaborating support groups and charities, namely the Muscular Dystrophy Association, Ataxia UK, HSP support group, PSP Association, HD Association of England and Wales were invited to join a stakeholder group. They attended an initial workshop (Workshop 1) to define conceptual considerations in relation to the physical activity interventions and outcomes in our target population, namely, people living with RNCs.

This was followed by a second stakeholder workshop (Workshop 2) with people living with RNC and the relevant charity representatives. They worked together to (a) explore issues and experiences relating to physical activity in the face of living with a RNC and (b) identify and priorities key constructs and domains of importance that would need to be measured when evaluating a physical activity intervention. Representatives from RNC charities were asked to gather views from their members living with RNCs prior to the meeting through their communication channels, e.g., surveys and social media platforms.

### Step Two: Finding Existing Outcome Measurement Instruments

We conducted a scoping review of systematic reviews published between January 2008 and December 2018 to identify outcome measures used to measure efficacy of any type of physical activity intervention for adults with neuromuscular diseases, motor neurone disease (MND), HD, PSP, multiple system atrophy (MSA), inherited ataxias and HSP (Open Science Framework registration: https://osf.io/4cr32/). The research team, experts in this field, were aware that little research into physical activity had taken place until the early 2000s and reviews came later, hence the 10-year window. Studies were included if participants were adults and if the reviews reported at least one outcome measure to evaluate the efficacy of the physical activity intervention at either the body structure/function, activity and/or participation levels, according to the International Classification of Functioning, Disability and Health (ICF) (20). Constructs and domains of importance identified during Workshop 2, were matched with outcome measures identified in the scoping review. Where no measures matched the identified, domains, additional literature searches were done using the domain descriptions as search terms. Additional criteria for selection were use in RNC or other neurological diseases. Two of the descriptors, motivation and confidence, relate strongly to self-efficacy so this was also added as a search term. Elicitation of stakeholder opinions during a series of virtual meetings was undertaken to supplement this process and to identify proposed outcome measures that were consistent with the domains of importance identified by the stakeholder group.

### Step Three: Quality and Feasibility Assessment of Outcome Measurement Instruments

Outcome measurement instruments that are included in a core outcome set should ideally be reliable and valid for use in the target populations (22). Feasibility of use is a further consideration. The rarity of the diseases being studied meant that the psychometric properties of the measurement tools we identified had not been examined in these conditions. We thus considered the psychometric properties of the tools as applied to more common long term, neurological conditions where there were indications of some common impairments. The evidence for each was collated for presentation at step four.

### Step Four: Reaching Consensus on the Proposed Core Outcome Set

The outcome measure instruments under scrutiny matching the agreed domains of importance were examined by individual researchers then presented to the wider research team for technical discussions of the psychometric properties through a series of video meetings. Following the video meetings, lists of items assessed within each instrument were sent to the stakeholder group via e-mail to elicit further reflection on their relevance to the constructs of importance. A final face to face stakeholder workshop (workshop 3) and consensus procedure was undertaken to agree on the instruments for each outcome to be recommended for inclusion in the core outcome set.

## Results

### Step One: Conceptual Considerations

People living with RNCs (workshop 1 *N* = 5; workshop 2 *N* = 3), carers (workshop 1 *N* = 1) and charity representatives (workshop 1 *N* = 5; workshop 2 *N* = 5) considered it important that measurement tools were able to detect outcomes across domains of (A) function and well-being and (B) participation in activities. In terms of (A), staying well, ensuring good sleep and maintaining positive mood were of highest priority whilst in relation to (B), the ability and confidence to take control and make choices along with normalization of participation and social engagement were important. Through further discussion, the stakeholder groups agreed that these aspects were well-centered around (i) physical well-being; (ii) psychological well-being and (iii) participation in day-to-day activities as the primary domains of meaningful importance. Relevant constructs within the physical domain were physical function and independence. Constructs in the psychological well-being domain were emotional well-being, mood, enjoyment, motivation for physical activity and confidence, whilst leisure activities, work and activity that matters (personal choice) were constructs of importance within the domain of participation.

### Step Two: Finding Existing Outcome Measurement Instruments

Database searches identified 5,435 articles, and, after removing duplicates, 4,433 were screened by titles and abstracts, leaving 62 articles for full-text eligibility assessment. They were screened and 27 were included in the scoping review (4, 5, 8, 12, 13, 23–44). The results of the scoping review will be presented in detail separately. Nearly 200 outcome measures assessing outcomes of structured physical activity interventions (Table 1) were identified within these 27 articles. Dosage, intensity and duration of training regimes were highly variable but typically involved strength training, aerobic and respiratory, functional training and combined programs with very few focusing on physical activity behavior change.

**Table 1:** Characteristics (including outcome measures utilized) reported in studies included in scoping reviews of physical activity interventions in RNCs.

**References**	**Research designs of included studies**	**Number of participants**	**Participant characteristics**	**Controls**	**Outcome measures**
Cup et al. (24)	2 studies using pre-post design	17	Male/female: 7/10 Mean Age (Y): Study 1: 52.4 (range: 28–67) Study 2: 62.5 (range: 39–83)	No controls	Performance and satisfaction/Muscle strength/Grip force and pinch grip (Grippit)/Fine motor control (Purdue Pegboard), Range of Motion (goniometer). Activities of daily living (interview with ADL-Taxonomy); Life satisfaction (modified Life Satisfaction Checklist)
Habers and Takken (27)	2 RCTs, 1 non-RCT, 9 uncontrolled studies	161	Male, *N* = 57; Females, *N* = 104 Mean Age (Y): range 40–68	Not reported	Disease activity (e.g., serum levels of creatine kinase, aldolase, cytokines etc.)/Muscle strength/Aerobic fitness/Functional performance/Functional capacity/Health status/Lung function/Muscle characteristics/Disease impact/Fatigue
Quinlivan et al. (13)	3 non-randomized studies	27	2 out of 3 studies reported gender: 9 males and 9 females in total. Mean Age (Y): range 32–61	Same training but in healthy controls, age- and sex-matched healthy, sedentary controls otherwise not specified	Borg rating of perceived exertion/VO2 max /HR/Superficial EMG for muscle activity and glucose and lactate blood levels/Serum creatine kinase /Respiratory gas exchange/Cardiac output and serum samples for lactate and glucose were measured/Needle muscle biopsy of vastus/Respiratory gas exchange data were collected/VO2 peak, and gross mechanical efficiency during the constant workload test/HR using 12 lead ECG tracing/Capillary blood samples lactate and ammonia
Ydemann et al. (28)	1 Pre-post cohort, 4 RCTs, 1 prospective cohort, 1 quasi-RCT, 1 descriptive study	757	Not reported	Standard medical therapy, usual care, daily interruption of sedation only, general physiotherapy alone	6 MWD/MIP/Isometric quadriceps force/Subjective feeling of functional well-being/Time in bed/ICU stay/Hospital stay/Duration of delirium/Ventilator-free days/Muscle fatigue and isotime dyspnoea/Atrophy/Weaning of atrophy (no further details provided)
Voet et al. (23)	4 RCTs and 1 quasi randomized study	170	20 adults with mitochondrial myopathy, diagnosed on the basis of clinical, familial and muscle biopsy data. 35 adults with myotonic dystrophy type 1, genetically confirmed. 36 adults with myotonic dystrophy (2 congenital form, 34 classical adult type), diagnosis not verified. 65 adults with FSHD, genetically confirmed. 9 adults with dermatomyositis and 5 adults with polymyositis.	Strength training vs. no training	Differences in Muscle strength (using dynamometer)/Quantitative Muscle Assessment fixed myometry testing system/Dynamic strength was evaluated using the one repetition maximum / Weight-lifting capacity/ Endurance time measured in a submaximal cycling test at a constant workload of 70%/ 6 MWT, VO2 max/ Maximum duration of contraction at 80% of MVIC on an isokinetic dynamometer/Sickness Impact Profile and the Symptom-Checklist /Nottingham Health Profile/ SF-36 Health Survey/ CIS-fatigue
Gianola et al. (25)	4 (3 controlled and 1 randomized clinical) trials. One study was excluded as included participants under 18 years old.	128	Mean Age of participants range from 22 to 48 years	No control/healthy control group/other interventions	Maximal voluntary isometric contraction /Maximal peak torque/Modified MRC/Six-Minute Bicycle Test/BORG/Six-minute walk test (m)/Timed-stands test/Timed up-and-go test/MVIC isokinetic torque/Test 80% MVC (sec)/Descending stairs/Climbing stairs/Standing up from a chair/Standing up from lying supine/Walking 6 min (comfortably)/Walking 50 m (fast) (sec)/CIS-fatigue/ICF functional dimensions
Narayanaswami et al. (26)	5 Class III studies	62	12 patients with Welander distal myopathy 9 ambulatory patients with LGMD2I and 9 healthy controls 11 men with BMD and 7 healthy men 8 patients with hIBM3 secondary to a defect in the MYH2 gene 6 patients with hIBM3 secondary to a defect in the MYH2 gene	Sedentary, age-matched controls	Maximal oxygen uptake/Maximal workload, and other patient-reported outcomes/Maximum workload/Muscle strength/Change in the expression of myosin isoforms on muscle biopsy
Khan and Amatya (32)	1 SRV, 1 RCT, 1 case-control, 5 prospective or retrospective cohort studies, 6 case series/reports	422	Not reported	Low-intensity home based program of maintenance exercises and education for self-management (30 min twice a week) (RCT) or healthy controls.	HRQoL/FIM/PIPPS/DASS/WHOQoL/LOS/Modified Barthel Index/MRS /HDS NHP/BI/ESS/HAS
Simatos Arsenault et al. (12)	1 single subject, 4 case reports, 1 quasi experimental design, 1 RCT	133	66 females vs. 67 males Mean Age (Y): 43.8	No exercise or lower intensity, home-based ambulatory exercise, otherwise not reported	FSS/Activity monitor/SF-36, FIS/Perceived mental functioning/Physical fitness (peak work levels, VO2 mL/min, mL/kg/min)/Ventilation/Isokinetic leg strength (total work capacity)/General mobility/Confidence in walking/Cardiorespiratory cycle ergometer test/Isokinetic muscle strength/Functional outcome of daily physical activity (RAM)/FIS (cognitive, physical, and social)/GBS disability score/HADS/RHS/QOL SF-36/MMT/WHOQOL-BREF/DASS-21/PIPP/Physical fitness (duration of exercise, distance walking, distance cycling, grip strength)/Pulmonary fitness (PEFR, FVC, FEV1)
Young et al. (29)	1 Randomized controlled single blind trial	29	Not reported	No strength training	Muscle strength voluntary contraction/Isokinetic knee torques/Timed functional activities
Sman et al. (5)	3 RCTs, 5 quasi-experimental (i.e., pre-post testing), 1 case report	134	Average age: 38 years old. 8 out of 9 studies reported gender: 52% were male, 48% female.	Where reported: controls underwent the same program, however, balance training was managed by a physiotherapist instead of a mechanical apparatus	Muscle strength (N or Kg)/Maximal voluntary isometric testing (Kg)/Isokinetic knee torque flex/extension/MVC/Endurance test at 80% MVC/Isokinetic muscle strength (Nm)/Medical research council scale (MRC)/BOT (balance) score/Power/Long jump (cm)/6 MWT/Walking ability (different parameters)/Functional activities (e.g., Chair raise)/CMTES/Phone FITT FDI/ROM/Tinetti Scale/Berg Balance scale/Physiological (BMI,FFM, Percent body fat, Serum myoglobin, RMS (μv)/Fatigue Severity Scale/Modified PCI/MHC/Myosin heavy chain/Cardiorespiratory cycle test/Mean blood CK/VAS/VO2 max/HR/Respiratory Borg Scale/METS/Fatigue Borg Scale
Corrado et al. (30)	4 RCTs and 1 Cohort	236	Not reported	No intervention	Quantitative neuromuscular assessment/Bioelectrical impedance analysis/6 MWT/MVC (myometer or isokynetic dynamometre)/Borg scale/Serum level of myoglobin/Surface electromyography techniques/Holter
Lui and Byl (33)	Prospective clinical studies (*N* = 2), RCTs (*N* = 2) and 1 SRV (Dalbello-Haas et al. (47), previous version of Dal Bello and Florence (4))	98 (including Drory et al. (45); Bello-Haas et al. (46)), excluding Dalbello-Haas et al. (47)	Not reported	Usual care/home exercise program without supervision/no exercise participation or usual activities	Norris ALS score strength/ALS-FRS strength MMT/FSS/FIM/FVC
Ng et al. (37)	3 prospective studies, 1 cross-sectional, pre-post case series	779	Not reported	General neurology clinic or general MND care	Survival, hospital readmissions and length of stay, SF-36, VAS on life satisfaction and well-being, ALSSS, ALSFRS, CSI, healthcare costs
Dal Bello-Haas and Florence (4)	2 studies, 6 and 12 month parallel group (1 randomized and 1 quasi randomized trial)	52	27 people with definite or probable, probable with laboratory-supported MND (El Escorial criteria), aged 41–80 years. Early stage MND. 25 people with definite or probable MND (El Escorial criteria), aged 41–80 years. Mild to moderate stages of MND.	The control condition was either no exercise or standard rehabilitation management (for example, range of motion exercise or stretching exercise).	LSFRS/the SF-36 to measure quality of life/FSS/Manual muscle strength testing
Eidenberger and Nowotny (34)	2 RCTs, 1 pre-experimental study and 1 with a historical control group	87	Male, *N* = 57; Females, *N* = 30 Mean Age (Y): range 53–63 years	Sham training/historical controls/no controls/ lowest possible load	Respiratory-related OMs (e.g., Spirom/FVC/MIP/MEP etc.)/Total survival time/6 MWT/Hand-held dynamometry/ ALSFRS/ FSS/HRSD/ESS/FIM/EQ-5D/SF-36/Chronic Respiratory Questionnaire
Arbesman and Sheard (36)	2 RCTs/2 non-RCTs + single subject study	287 (including Dal Bello-Haas and Florence (4))	Only one study (i.e., single-case study) reported: one male, age 62 years. Drory et al. (45) and Bello-Haas et al. (46) and already presented in previous systematic reviews.	Training vs no training/or general care	ALSFR/ Medical Outcomes Survey 36-item/QoL: SF−36/Life satisfaction and well-being/visual analogue scales ROM/Muscle strength and shortness/Grip strength/Functional activities—Modified Norris Limb Scale/Muscle strength measured with Chatillon push–pull gauge/Survival
Ferreira et al. (35)	3 RCTs	63	Not reported	Comparison with controls who had not received RMT full time or were receiving training without load	Ventilatory function FVC, FEV1, MVV/Respiratory muscle strength, MEP and MIP)/Functional capacity, 6 MWT
Quinn and Busse (38)	4 studies with different designs: before/after design (*N* = 1), single case (*N* =1), observational (*N* = 1), RCT (*N* =1)	63	Male: female = 17:23 (only reported in 1 study - Zinzi et al. (48)). Age not reported except for one single case study = one male, 49 years old	Healthy controls/healthy matched controls/usual care (pharmacological)	Range of motion/Flexibility/Strength/Co-ordinated and reciprocal movement/Standing, one foot and kneeling balance/Breathing volume and control/SF-36/Number of falls/Modified falls scale/Berg Balance Scale/Self' paced/Fast paced gait speed/UHDRS/Physical examinations of posture/Zung depression scale/MMSE/Barthel Index (ADL)/Tinetti scale (balance)/PPT/Rehabilitation evaluation scale (REHAB)/BMD/Interact (behavior assessment)/HR/BP/RR/SHRS
Fritz et al. (39)	2 Observational (without control), 6 RCTs, 7 Pre-Post control group studies, 2 Pseudo RCT, 1 single case study	435	Male = 47.25% Mean Age (Y): range 28–57	Usual care (*N* = 2), no progression in resistance training (*N* = 1), sham (*N* = 1), otherwise not specified	Balance/Fitness (cardiovascular function)/Goal attainment/Motor function and performance/Muscle strength/Number of falls/Physical activity/Pulmonary function/Rate of chest infections/Ulcer staging/Spatiotemporal and kinematic parameters of gait and balance/Walking ability and endurance/Outcome measures of cognitive function included cognition and psychological measures (depression, anxiety, and apathy)
Koopman et al. (31)	3 RCTs (2 included in the scoping review)	120	One study was conducted in elderly people (no details provided). No details are reported in the other studies	No treatment or usual care	Self perceived activity limitations (e.g., Physical Component Summary of the SF-36 PCS/Physical mobility category of the Nottingham Health Profile)/Muscle strength/Muscle endurance fatigue/Pain/Adverse events subdivided into minor adverse events and serious adverse events
Trujillo-Martín et al. (40)	1 Clinical trial (pre-post design)	87	Mean age (Y), (SD) = 38.1 (10.9)	No controls	Neurological examination using the Romberg's Test and a coordination test with a computer
Fonteyn et al. (41)	14 prospective clinical trials (4 moderate quality i.e., comparative studies - 1 on cerebellar ataxia).	84	Not reported	Controls were patients receiving treatments later or not specified	Balance/gait/muscle strength/range of motion/ataxia severity/fall frequency/gait speed/ADL/FIM/Barthel Incapacitation scores/Hamilton Rating Scale for Depression/WHOQOL-BREF/NESSCA/SARA
Marquer et al. (43)	19 studies including MS and traumatic causes. In this scoping review only 4 were included: 1 RCT and 3 observational studies. However, 3 of these were already included in Fonteyn et al. (41). Only Foltz and Sinaki (49) is described in the scoping review.	19	Not reported	No controls	Subjective self-evaluation of balance
Milne et al. (42)	4 RCTs 1 Pseudo-RCT 4 Interrupted time series without a parallel control group 5 Case series	292 in total (21 were not adults) = 271	Mean age range (Y): 23.3–62.5 A total of 228 participants (out of 292) were ambulant, and 72 were non-ambulant. In 2 studies, ambulation status was not reported	No controls or pharmacological management alone or control group completing verbal health education and upper limb exercises (compared to a cycling regime) or a control group receiving sham vibration over the same duration (compared to stochastic vibration)	SARA/FIM/Gait speed/ cadence/ FAC/ Number of falls/ ICARS (8 items)/10 mWT/ Gait speed/ Standing capacity/ Spread of feet/ Body sway/ Knee to tibia test/ Action tremor/ SF-36/EQ-5D/EQ-VAS/ABC/SCAFI/INAS/GAS/BBS/Kinematic and kinetic gait parameters/Static balance test/ Dynamic balance acceleration treadmill task/DGI/ TUG/ FRT/ ABC/ Sway amplitude/ Spatiotemporal/ gait parameters/ FES-I LOS/ SOT/CoP area of 95% confidence/Ellipse CoP sway path/ CoP mean velocity/ Barthel WHOQOL-BREF/ MBI/ 5-item Barthel Index/ Obstacle avoidance task on a treadmill/ EFAP obstacle subtask/ Sway area
Hajjar and Cooper (44)	2 quasi randomized controlled trials	38 (two studies based on the same sample)	Not reported	Balance exercises only	Kinematic gait measures (stance time, swing time, and step length)/2.4-m walk test/Timed “Up & Go” Test/Vertical Gaze Fixation Score/Gaze Error Index
Intiso et al. (8)	6 case reports, 3 case series, one case-control study, one quasi randomized trial and one randomized controlled trial	88	Gender (number of F/M), information only in 3 studies: Case series 1: 6/2 Case series 2: 3/7 Case series 3: 2/3	Balance exercises only [same studies as in (44)]	BBS/ ABC Scale/Sharpened Romberg Test/ FRT/360 turns/ TUG test/ 6-MWT/10-WMT/15.2-meter walk test/8-foot (2.4-me) walk test/5-step test/Balance and gait parameters/ABF device/Static and dynamic baropodometry/Computerized systems including the GAITRite system/3D-GA/Force platforms/PSPRS/UPDRS

*6 MWT, Six Minute Walking Test; RM, Repetition Maximum; VO2 max, Maximal Oxygen uptake; 6 MWD, Six Minute Walking Distance; ICU, Intensive Care Unit; CIS-fatigue, Checklist Individual Strength; MVC, Maximum Voluntary Contraction; MVIC, Maximum Voluntary Isokinetic Strength; PIPPS, Perceived Impact of Problem Profile Scale; ESS, Environmental Status Scale; CK, Serum creatine kinase; ALS, Amyotrophic Lateral Sclerosis; ALS-FRS, Amyotrophic Lateral Sclerosis Functional Rating Scale; EQ-VAS, EQ–Visual Analogue Scale; DGI, Dynamic Gait Index; LGMD1, Limb-girdle muscular dystrophies autosomal dominant; LGMD2, Limb-girdle muscular dystrophies autosomal recessive; MND, Motor Neurone Disease; RCT, Randomized Controlled Trial; PNF, Proprioceptive Neuromuscular Facilitation; SRV, Systematic Review; ALSFRS, Amyotrophic Lateral Sclerosis Functional Rating Scale; MT, Manual Muscle strength Testing; FSS, Fatigue Severity Scale; FIM, Functional Independence Measure; FVC, Forced Vital Capacity; BiPAP, Biphasic Positive Airway Pressure; MIP, Maximum Inspiratory Pressure; MEP, Maximal Expiratory Pressure; HRSD, Hamilton Rating Scale for Depression; ESS, Environmental Status Scale; EQ-5D, Health Status questionnaire; SF-36, Short Form-36 Health Survey; SNIP, Sniff Nasal Inspiratory Pressure; RMT, Respiratory Muscle Training; FEV1, Forced Expiratory Volume; MVV, Maximum Voluntary Ventilation; QoL, Quality of Life; CMTES, Charcot-Marie-Tooth; Examination Score; FITT, Frequency-Intensity-Time-Type; ROM, Range of motion; BMI, Body mass Index; FFM, Fat Free Mass; RMS, Root Mean Square; PCI, Physiological Cost Index; MHC, Myosin Heavy Chain; VAS, Visual Analogue Scale; METS, Metabolic Equivalent of Task; UHDRS, Unified Huntington's Disease Rating Scale; MMSE, Mini Mental Status Examination; PPT, Physical Performance Test; REHAB, Rehabilitation Evaluation Scale; BMD, Behavior and Mood Disturbance scale; HR, Heart Rate; BP, Blood pressure; RR, Respiratory Rate; SHRS, St Hans rating scale; ADL, Activity Daily Living; LOSWHOQOL-BREF, World Health Organization Quality of Life questionnaire; NESSCA, Examination Score for Spinocerebellar Ataxia; SARA score, Scale for Assessment and Rating of Ataxia; FAC, Functional Ambulation Classification; ICARS, International Cooperative Ataxia Rating Scale; ABC, Activities specific Balance Confidence; SCAFI, Spinocerebellar Functional Index; INAS, Inventory of Non-Ataxia Signs; GAS, Goal Attainment Score; BBS, Berg Balance Scale; TUG, Timed up-and-go test; FES, Falls Self-Efficacy Scale; LOS, Limits of stability; SOT, Sensory Organization Test; WHOQOL-BREF, WHO Quality of Life-BREF; EFAP, Emory Functional Ambulation Profile; RAM, Rotterdam Activity Monitor; FIS, Fatigue Impact Scale; HADS, Hospital Anxiety and Depression Scale; RHS, Rotterdam Handicap Scale; MMT, Manual muscle testing; DASS-21, Depression, Anxiety; Stress scale (short form); PIPP, Perceived Impact of Problem Profile; PEFR, Expected Peak Expiratory Flow Rate; FEV1, Forced Vital Capacity in 1 s; HRQoL, Health-related Quality of Life; MBI, Modified Barthel Index; MRS, Modified Rankin Scale; HDS, Hughes Disability Scale; NHP, Nottingham Health Profile; BI, Barthel Index; HAS, Handicap Assessment Scale; ALSSS, Amyotrophic Lateral Sclerosis Severity Scale; CSI, Caregiver Strain Index; BBS, Berg Balance Scale; ABC, Scale Activities-specific Balance Confidence; FRT, Functional Reach Test; 10-WMT, Ten-Meter Walk Test; ABF, Audio-biofeedback device; 3D-GA, 3D-Gait Analysis; PSPRS, Progressive Supranuclear Palsy Rating Scale; UPDRS, Unified Parkinson's Disease Rating Scale; FSHD, Facioscapulohumeral Muscular Dystrophy; CoP, Centre of Pressure; OMs, Outcome Measures; FITT FDI, frequency, duration, and intensity score for the Phone-FITT scale*.

We mapped each outcome to the World Health Organisation International Classification of Function (ICF) domains (20) (see Table 2). The majority were related to function and activity. Outcomes reflective of both body structure impairments and participation were less frequently reported. Two domains were categorized as “Other” (e.g., Goal attainment score), and “Disease-specific” questionnaires (e.g., Unified Huntington's Disease Rating Scale or Scale for the Assessment and Rating of Ataxia).

**Table 2:** Mapping to World Health Organisation international classification of function ICF) domains.

**References**	**Condition included**	**Body-structure**	**Body-function**	**Activity**	**Participation**	**Disease-specific**	**Other**
Voet et al. (23)	Muscle diseases (myotonic dystrophy, polymyositis and dermatomyositis, facioscapulohumeral muscular dystrophy, mitochondrial myopathy)		✓		✓		
Cup et al. (24)	Myotonic dystrophy and Welander distal myopathy		✓	✓	✓		
Gianola et al. (25)	Muscular dystrophy		✓	✓			
Narayanaswami et al. (26)	Welander distal myopathy, Becker muscular dystrophy, Limb-girdle muscular dystrophies, Hereditary inclusion body myopathies	✓	✓				
Habers and Takken (27)	Idiopathic inflammatory myopathy (dermatomyositis, polymyositis, and inclusion body myositis)	✓	✓		✓		
Ydemann et al. (28)	Critical illness myopathy and polyneuropathy		✓	✓	✓		✓
Young et al. (29)	Charcot-Marie-Tooth disease		✓	✓			
Sman et al. (5)	Charcot-Marie-Tooth disease	✓	✓	✓		✓	
Corrado et al. (30)	Charcot-Marie-Tooth disease	✓	✓	✓			
Quinlivan et al. (13)	McArdle disease	✓	✓				
Koopman et al. (31)	Postpolio syndrome		✓	✓			✓
Simatos Arsenault et al. (12)	Guillain-Barré Syndrome		✓	✓	✓	✓	
Khan and Amatya (32)	Guillain-Barré Syndrome		✓	✓	✓		
Dal Bello-Haas and Florence (4)	Motor-neuron disease		✓		✓	✓	
Lui and Byl (33)	Motor-neuron disease		✓			✓	
Eidenberger and Nowotny (34)	Motor-neuron disease		✓	✓	✓	✓	
Ferreira et al. (35)	Motor-neuron disease		✓	✓			
Arbesman and Sheard (36)	Motor-neuron disease		✓		✓	✓	
Ng et al. (37)	Motor-neuron disease				✓	✓	✓
Quinn and Busse (38)	Huntington's disease		✓	✓	✓	✓	
Fritz et al. (39)	Huntington's disease		✓	✓			✓
Trujillo-Martín et al. (40)	Spinocerebellar ataxia		✓				
Fonteyn et al. (41)	Cerebellar Ataxia		✓	✓	✓	✓	
Milne et al. (42)	Genetic degenerative ataxia		✓	✓	✓	✓	✓
Marquer et al. (43)	Cerebellar Ataxia		✓				
Hajjar and Cooper (44)	Progressive supranuclear palsy		✓	✓			
Intiso et al. (8)	Progressive supranuclear palsy		✓	✓		✓	✓

Eleven reviews utilized disease-specific outcome measures, while in six reviews measures were not able to be represented within the ICF domains (i.e., in the “Other” category). Most studies (*n* = 17) included outcomes that were representative of three to five different domains. Notably, there was no evidence of stakeholder engagement or involvement of people with the condition being investigated in the selection of measures used as primary outcomes.

Constructs relevant to the *physical and psychological well-being*, and *participation in day-to-day activities* domains were cross-checked with the outcomes synthesized in the scoping review. No single outcome measurement instrument that addressed all three domains was identified. Measures were usually tailored to specific activities or functions (e.g., on lower limb function, ability to perform daily tasks). Alternative outcomes reflective of well-being were reviewed by stakeholders through a series of group discussions. None of these comprehensively matched the domains of importance identified in Step One.

Further literature searching resulted in the Oxford Participation and Activities Questionnaire (Ox-PAQ) (50, 51) being identified as an instrument that matched the majority of the constructs highlighted as relevant in Step One and has been used in one RNC disease group and other neurological conditions (50). Three constructs (i.e., enjoyment, motivation and confidence) are important predictors of physical activity behavior and not assessed within any of the Ox-PAQ items but are relate highly to self-efficacy. The Sources of Self-Efficacy for Physical Activity (52) was thus identified as an additional secondary outcome able to reflect these constructs. Other self-efficacy scales found in the search were specific to particular diseases and populations but did not include neurological conditions. It is important to note that these outcomes were not only reflective of that which is important to stakeholders but also additionally are able to provide mechanistic insight for researchers.

### Step Three: Quality and Feasibility Assessment of Proposed Outcome Measurement Instruments

The OxPAQ questionnaire is a short, 23-item, patient-reported outcome measure, that has been specifically developed for cross-disease application and validated in three long term neurological conditions (MND, Parkinson's disease, Multiple Sclerosis) (50). It was developed using patient interviews and expert reviews and has a manual and online scoring. The Ox-PAQ reports on three domains, Routine Activities (14 items), Emotional Well-Being (5 items) and Social Engagement (4 items). Routine Activities assesses individuals' capacity to engage in regular activities that form the basis of daily life. Emotional Well-Being provides an indication of current mental health status, while Social Engagement assesses whether individuals can maintain relationships, both personal and from a wider community perspective. Internal reliability is high (Cronbach's α 0.81–0.96) and validity was demonstrated against relevant domains of the MOS SF-36 and the EQ-5D-5L (50). Sources of Self-Efficacy for Physical Activity is an 18-item questionnaire that measures six aspects (3 items for each source) of self-efficacy for physical activity, specifically: mastery experience, vicarious experience, verbal persuasion by others, self-persuasion, negative affective states and positive affective states (52). Items were pooled from prior qualitative studies, scales of feelings induced by physical activity and sources of self-efficacy more generally. It was refined in a study of 1,406 German adults through principal axis analysis with inter-related factors and confirmatory factor analysis. It is a reliable (Cronbach's α 0.75–0.93), valid (convergent and discriminant). It has not been validated in neurological populations, but the scale was designed to be generally inclusive allowing it to be applied across conditions and populations (52). Other self-efficacy scales target specific conditions and were not generalizable or applicable to people with RNC.

### Step Four: Reaching Consensus on the Proposed Core Outcome Set

Following broad group communication and discussions and a final face to face consensus procedure involving small group discussions, it was agreed that the Ox-PAQ and the Sources of Self-Efficacy for Physical Activity measure should be assessed in trials evaluating physical activity interventions across RNCs given the ways in which they matched the domains and constructs of importance identified by the stakeholder group. This was a group decision by people living with RNC, charity representatives and the research team at the final workshop.

## Discussion

Physical activity research trials in RNCs to date have typically involved targeted exercise intervention and evaluation at the specific disease level despite these diseases leading to variable but similar impairments and functional impacts (for example fatigue, muscle weakness, balance problems, falls and difficulty walking). Our scoping review highlighted the prevalence of interventions, mainly focusing on structured exercise and typically underpinned by standard approaches (53) highlighting the role of physical activity and exercise as a critical enabler of participation for all those living with common and rarer long term neurological diseases (54). Our scoping review of the literature identified outcome measures appropriate for the specific body structure, function and activity level changes targeted by these interventions, but there was a degree of mismatch between these outcomes and constructs identified as important to people living with RNCs (e.g., assessments that capture changes at the level of participation).

We utilized a person-centered approach leading to the proposal of a meaningful core outcome measurement set for use when researching physical activity interventions for people living with RNCs. Our collaborative and participatory design involved members of the public, including people living with RNCs, representatives of charities and support groups for RNCs and is the first core outcome set to our knowledge which has specifically focused on physical activity interventions for RNCs. Stakeholder engagement is receiving increasing recognition in patient-reported outcomes research (18) and clinical trials (55, 56) so as to ensure that interventions and outcomes are relevant to the target populations. A core outcome set for disease modification trials for dementia has been developed with stakeholder input and involvement of the research community. This was achieved through a number of stages, including a systematic review of outcome measures, a consultation with patient and public involvement representatives and a final consensus reached with the dementia research community (20). A similar approach was used to develop a core outcome measure set for exercise studies in Multiple Sclerosis (57), where a group consisting of experts in the field, support group representatives and expert patients, jointly discussed a pre-defined core set for Multiple Sclerosis. This was based on the World Health Organisation International Classification of Function and included body structure and function, activity and participation categories. Our approach differed somewhat in that we initially elicited discussion and reflection from our stakeholder groups on the domains considered important when engaging in physical activity interventions, but without presenting any work undertaken in previous studies.

Outcomes identified in the scoping review assessed the effect of physical activity interventions primarily at the level of body functions and structures, functional activities. There were fewer identified outcomes at participation level, in contrast to the domains prioritized by our stakeholder group, namely physical and psychological well-being and participation to day-to-day activities. In the scoping review, measures of quality of life and health-related well-being were identified, but these did not (in the views of our stakeholder group) sufficiently capture the breadth of areas of importance in relation to participation and physical activity in RNCs. For example, the 36-Item Short Form Survey is more focused on levels of vigorous and moderate activities, rather than independence in day-to-day activities. The Ox-PAQ and the Sources of Self-Efficacy for Physical Activity measure were however considered to reflect meaningful outcomes of physical activity interventions for people with RNCs.

Whilst the identified and proposed outcomes are clearly relevant to people with RNC, it is not yet clear how well the measures perform within and between these populations nor whether they fully capture that which is meaningful to people with RNCs. For example, the Sources of Self-Efficacy Scale may not fully capture enjoyment for physical activity; it may be that a purpose developed enjoyment scale (58, 59) is more appropriate in different settings. The broad range of rare neurological diseases where physical activity interventions are indicated are a specific challenge. A key limitation is that we did not consistently have stakeholders present at all workshops with faster progressing conditions, those with significant cognitive disorders or carers, relying on the charity representatives to bring accounts of these experiences. People were invited, but the additional complexity of those conditions may have affected engagement in all steps. Future validation work will need to include these groups to inform the implementation of the proposed core outcome set.

## Conclusion

We propose a core outcome set, developed in collaboration with people living with RNC and their representatives, for use in studies of physical activity interventions. The two measures proposed were selected to include domains of importance to people living with these diseases.

## Data Availability Statement

The original contributions presented in the study are included in the article/supplementary material, further inquiries can be directed to the corresponding author/s.

## Ethics Statement

Ethical review and approval was not required for the study on human participants, in accordance with the local legislation and institutional requirements.

## Author Contributions

GR and MB: conception and organization of the research project, design and review and critique of the analysis, and writing of the first draft and review and critique of the manuscript. VB: organization of the research project, design and review and critique of the analysis, and writing of the first draft and review and critique of the manuscript. HD: conception and organization of the research project, design of the analysis, and writing of the first draft and review and critique of the manuscript. AB, TJ, JM, LP, RP, MR, and LR: conception of the research project, design of the analysis, and review and critique of the manuscript. FJ and ER: conception and organization of the research project, design of the analysis, and review and critique of the manuscript. All authors contributed to the article and approved the submitted version.

## Funding

This work was funded by an NIHR Programme Development Grant RP-DG-0517-10002 (Co-Chief Investigators: GR and MB). This is a summary of independent research funded by the National Institute for Health Research (NIHR)'s Programme Development Grant Programme. Centre for Trials Research receives funding from Health and Care Research Wales and Cancer Research UK. Open Access funding has been made available from Cardiff University.

## Author Disclaimer

The views expressed are those of the authors and not necessarily those of the NHS, the NIHR, or the Department of Health and Social Care.

## Conflict of Interest

The authors declare that the research was conducted in the absence of any commercial or financial relationships that could be construed as a potential conflict of interest.

## Publisher's Note

All claims expressed in this article are solely those of the authors and do not necessarily represent those of their affiliated organizations, or those of the publisher, the editors and the reviewers. Any product that may be evaluated in this article, or claim that may be made by its manufacturer, is not guaranteed or endorsed by the publisher.
